# Reevaluating nutrition as a risk factor for cardio-metabolic diseases

**DOI:** 10.25100/cm.v49i2.3840

**Published:** 2018-06-30

**Authors:** Patricio López-Jaramillo, Johanna Otero, Paul Anthony Camacho, Manuel Baldeón, Marco Fornasini

**Affiliations:** 1 Dirección de Investigaciones Fundación Oftalmológica de Santander, Floridablanca, Colombia; 2 Facultad de Salud, Universidad de Santander, Bucaramanga, Colombia; 3 Facultad de Ciencias de Salud, Universidad Autónoma de Bucaramanga, Floridablanca, Colombia; 4 Facultad de Ciencias de la Salud Eugenio Espejo, Universidad Tecnológica Equinoccial, Quito, Ecuador

**Keywords:** nutrition, cardiovascular diseases, intake, legumes, nutrición, enfermedades cardiovasculares, ingesta, leguminosas

## Abstract

**Introduction:**

The consumption of saturated fats is considered a risk factor for cardiovascular diseases.

**Objective:**

Review published papers on the role of macro-nutrient intake in cardiovascular risk.

**Results:**

Recent reports from the PURE study and several previous meta-analyses, show that the consumption of total saturated and unsaturated fat is not associated with risk of acute myocardial infarction or mortality due to cardiovascular disease. High carbohydrate intake was associated with the highest risk of total and cardiovascular mortality, while total fat consumption or of its different types was associated with a lower risk of mortality. A high consumption of fruits, vegetables and legumes was associated with lower risk of total mortality and non-cardiovascular mortality. The consumption of 100 g of legumes, two or three times a week, ameliorated deficiencies of the nutrients contained in these foods and was associated with a reduction in the risk of developing chronic non-communicable diseases.

**Conclusion:**

A healthy diet should be balanced and varied, be composed of a proportion of complex carbohydrates rich in fibber between 50-55% of the daily energy consumed, of saturated and unsaturated fat (25-30%), animal and vegetable protein (including legumes) between 15-25%, vitamins, minerals and water. These nutrients are abundantly present in fruits, vegetables, cereals, legumes, milk and its derivatives, eggs and meats, so public policies should promote the availability and access to these nutrients within primary prevention programs to reduce the growing prevalence of cardio-metabolic diseases.

## Resumen

**Introducción:**

El consumo de grasas saturadas es considerado como un factor de riesgo para enfermedades cardiovasculares.

**Objetivo:**

Revisar trabajos publicados sobre el papel de la ingesta de macro-nutrientes en el riesgo cardiovascular.

**Resultados:**

Varios meta-análisis y reportes del estudio PURE demuestran que el consumo de grasa total, saturada e insaturada, no se asoció con riesgo de infarto agudo de miocardio o mortalidad por enfermedad cardiovascular. La alta ingesta de carbohidratos fue la que se asoció con mayor riesgo de mortalidad total y cardiovascular, mientras que el consumo de grasa total o de sus diferentes tipos se asoció con menor mortalidad. Un alto consumo de frutas, vegetales y legumbres se asoció con menor riesgo de mortalidad total y mortalidad no cardiovascular. El consumo de 100 g de leguminosas, dos o tres veces por semana contribuyó a mejorar las deficiencias de nutrientes contenidos en estos alimentos y está asociado con una disminución del riesgo de desarrollar enfermedades crónicas no transmisibles.

**Conclusión:**

Una dieta saludable debe ser equilibrada y variada, estar compuesta con una proporción de carbohidratos complejos ricos en fibra entre 50-55 % de la energía diaria consumida, de grasa saturada e insaturada (25-30 %), proteína animal y vegetal (incluidas las leguminosas) entre 15-25 %, vitaminas, minerales y agua. Estos nutrientes están abundantemente presentes en frutas, vegetales, cereales, leguminosas, leche y sus derivados, huevos y carnes, por lo que las políticas públicas deben promover la disponibilidad y acceso a estos nutrientes dentro de los programas de prevención primaria para disminuir la creciente prevalencia de enfermedades cardio-metabólicas.

## Introduction

The official concept that saturated fats cause coronary disease comes from 1961 when the American Heart Association (AHA) published the first recommendation to avoid the consumption of saturated fats, as well as cholesterol in order to prevent cardiovascular disease (CVD) ^1^. This proposal is known as the "hypothesis of the diet-heart relationship" and appeared as a necessary explanation to understand the risk of suffering from these diseases, which is of great interest both in public health and in clinical practice given the increased prevalence in recent years. However, the causality of this hypothesis has yet to be proven.

Cardiovascular disease is the leading cause of mortality worldwide, even in low- and middle- income countries such as Colombia and Ecuador ^2^. In Colombia, according to data from the Institute for Health Metrics and Evaluation, coronary ischemic disease remains the leading cause of mortality, with an increase of 21.26% from 2005 to 2016, while mortality from stroke has remained stable with a tendency to decrease minimally (-1.3%) ^3^. The importance of this topic to public health policy and in clinical practice, has led to a debate with controversial arguments, in the scientific literature and in specific articles such as the Teicholz and Thorn ^4^, with the arguments presented by these authors discussed in this review.

The objective of this review, and in particular of reference to the data from the Prospective Urban and Rural Epidemiology (PURE) study, is to demonstrate that a healthy diet should be balanced and varied. This refers to a diet composed of an adequate proportion of complex carbohydrates rich in fiber between 50-55% of the daily energy consumed, of saturated and unsaturated fat (25-30%), of animal and vegetable protein (including legumes) between 15-25%, of vitamins, minerals and water. Therefore, dietary recommendations to highly restrict the consumption of foods rich in cholesterol and saturated fats, while excessive consumption of especially of processed carbohydrates, should be re-evaluated since the consumption of foods rich in cholesterol have an impact on the plasma levels of LDL-cholesterol, but also on the concentrations of HDL-cholesterol, triglycerides and apolipoproteins. Moreover, studies in which the consumption of saturated fatty acids has been replaced by monounsaturated or polyunsaturated fatty acids from vegetable oils have not consistently shown a beneficial effect on the incidence of cardiovascular events and on the development of type 2 diabetes mellitus (DM2), which has been referred to as cardio-metabolic diseases.

## Substitute studies of saturated fats by vegetable oils

The aforementioned authors ^4^ affirm that during between sixties and eighties, trillions of dollars were invested in large clinical studies that included between 10,000 and 53,000 individuals in which different diets were tested in which saturated fats were replaced with vegetable oils rich in unsaturated fats. The results however, did not as expected support the proposed causative role of saturated fats in the presentation of CVD. The cited authors argue that many of these results were not disseminated, even one of the largest clinical trials, funded by the National Institutes of Health of the United States (NIH), which obtained results contrary to the expected beneficial effects of substitution of saturated fats for oils rich in unsaturated fat, which is why they were not published for more than 16 years ^5^.

Recently, several US publications reviewed the results of some clinical trials ^6^ ^,^ ^7^ that had been condemned to oblivion or reanalyzed ^7^. In none of the reviews conducted and in the meta-analyzes published on this subject, was it possible to document evidence that saturated fats had a negative effect on total mortality or cardiovascular mortality ^8^ ^-^ ^16^. Despite this evidence, dietary guidelines from different countries, which base their recommendations on US guidelines, indicate that the consumption of saturated fats should be less than 10% of the daily calories consumed. The recommendations of the AHA are even more restrictive, limiting the consumption of saturated fats to only 5-6% of the daily calories that should be consumed by individuals with high blood cholesterol levels ^17^ ^,^ ^18^.

The Presidential Council of the AHA in its most recent publication ^19^ concluded that replacing saturated fats with certain vegetable oils (soy, sunflower) reduces the risk of CVD by approximately 30%, a reduction that is only similar to that reported in clinical trials who used statins. However, Teicholz and Thorn ^4^ emphasize that these results are contrary to those of four reviews carried out by different authors ^9^ ^-^ ^13^, in which the estimated reduction in CVD risk was less than 19%. In addition, they noted that in two of these reviews statistical significance was lost when the authors used more rigid criteria and did not include poorly controlled clinical trials in the analysis. According to the authors cited ^4^, if only the results with statistical significance of well controlled clinical studies are examined, only two reviews agree with the results obtained by the AHA, while all the other analyzes give contrary results. According to Teicholz and Thorn ^4^ the contradictory findings in publications that have performed the analyzes of the same works independently can be explained by the criteria of the outcomes used. In seven reviews that used hard outcomes such as acute myocardial infarction (AMI), cerebrovascular accident (CVA), cardiovascular death or total mortality, it was found that replacing saturated fats with vegetable oils rich in polyunsaturated fatty acids, led to no beneficial effect. When a softer outcome was used, as the AHA does, such as the composite of several CV events, which also includes more subjective events such as angina, there is a small beneficial effect of the substitution, as well as if the analysis was based on selected studies. Teicholz and Thorn ^4^ argue that there is evidence that clearly questions the diet-heart relationship hypothesis.

## Contributions of the PURE study to clarify the role of macronutrients consumption in cardiovascular risk

Taking into account the series of contradictions in the mentioned publications, in the PURE study we evaluated the impact of the composition of the diet on certain risk factors such as lipid profile, blood pressure and CVD risk ^20^ ^-^ ^22^. More than 135,000 individuals from 18 high income (HIC), medium-income (MIC) and low-income (LIC) countries from five continents were followed for an average of 7.4 years during which we studied the association between macro-nutrient consumption and cardiovascular morbidity and mortality. In the first report ^20^, we showed that a higher consumption of fats (saturated, monounsaturated and polyunsaturated) and proteins of animal origin, was associated with a lower mortality rate, while the higher consumption of carbohydrates was associated with a increased mortality rate. The primary outcomes studied were mortality from any cause and major cardiovascular events: cardiovascular death, non-fatal MI, nonfatal stroke and heart failure. The secondary outcomes were all cardiac infarctions, stroke, cardiovascular mortality and non-cardiovascular mortality. The participants were categorized by quintiles of nutrient intake: carbohydrates, fats and proteins, based on the percentage of energy provided by each macronutrient. We evaluated the association between carbohydrate consumption, total fat and each type of fat with CVD and total mortality. We calculate the risk ratios (HR) using a Cox proportional hazards model. During follow-up, 5,796 deaths and 4,784 major CV events occurred. The highest consumption of carbohydrates was associated with an increase in the risk of total mortality (higher quintile 5 vs lower quintile 1) HR of 1.28; 95% CI: 1.12-1.46, *p* trend = 0.0001), but not with the risk of CVD or cardiovascular mortality. The intake of total fat and of each of its types was associated with a lower risk of mortality from any cause (quintile 5 vs. quintile 1 of total fat: HR 0.77; CI 95%: 0.67-0.87; *p* trend <0.0001, of saturated fat; HR 0.86; CI95%: 0.76-0.99, *p* trend = 0.0088, of monounsaturated fat; HR 0.81; 95% CI: 0.71-0.92, *p* trend <0.0001, and polyunsaturated fat; HR: 0.80 [95% CI: 0.71-0.89] p trend <0.0001). A higher intake of saturated fat was associated with a lower risk of stroke (quintile 5 vs. quintile 1, HR 0.79, 95% CI 0.64-0.98, *p* trend = 0.0498). The consumption of total, saturated and unsaturated fat was not associated with risk of AMI or mortality due to CVD. In conclusion, our findings show that high carbohydrate intake is associated with greater risk of total mortality, while the consumption of total fat or its different types is associated with lower mortality from any cause and is not associated with CVD, AMI or CV mortality. In addition, the consumption of saturated fat had an inverse association with CVA.

These results show that in the studied population, the majority of which were from LIC’s and MIC’s, there is an inverse relationship between meat and milk consumption (which were the main source of proteins and of saturated and monounsaturated fats) and all-cause mortality. This finding may be related to the fact that animal products are rich in zinc, iron, vitamin K2 and vitamin B12, micronutrients that are deficient in LIC and MIC populations ^23^. In these countries, there is a low consumption of animal products because they are expensive and inaccessible ^24^, determining a high relative consumption of carbohydrates in a compensatory way, which, as indicated, was associated with higher mortality. Also, it could be that a diet with an adequate supply of proteins and fats, would reduce age related loss of muscle mass and strength and the risk of sarcopenia, low strength has been identified as a CVD risk factor ^25^ in the same cohort (also the PURE study).

## Consumption of fruits, vegetables and legumes and cardiovascular risk

In the second article ^21^, we reported that adequate intake of fruits, legumes and raw vegetables, foods that are rich in complex carbohydrates, is associated with lower overall mortality. This apparent discrepancy suggests that it is the high consumption of processed carbohydrates (sugar and refined flours) that is associated with the highest mortality observed in the individuals studied. To assess consumption, we used a food frequency questionnaire specific to each country and standardized questionnaires to collect information on demographic variables (age, sex), socio-economic (education, income, employment), lifestyles (smoking, physical activity, intake of alcohol), clinical history, use of medications and family history of CVD. The main outcome was the presence of CVD and, as in the previous publication; we used the same statistical analyses to evaluate the association between consumption of fruits, vegetables and legumes with the risk of CV events and total mortality. The average consumption of fruits, vegetables and legumes was 3.91 (SD 2.77) servings per day. A greater total consumption of fruits, vegetables and legumes was inversely associated with CVD, AMI, CV mortality, non-CV mortality and total mortaliy, in the model adjusted for age, sex and medical center. The estimates were attenuated in the multivariate model. CVD; HR: 0.90; 95% CI: 0.74-1.10, *p* trend= 0.1301, AMI; HR: 0.99; 95% CI: 0.74-1.31, *p* trend= 0.2033, ACV; HR: 0.92; 95% CI: 0.67-1.25, *p* trend= 0.7092, CV mortality; HR: 0.73; 95% CI: 0.53-1.02, *p* trend= 0.0568, non-CV mortality; HR: 0.84; 95% CI: 0.68-1.04, *p* trend= 0.0038, and total mortality; HR: 0. 81; 95 % CI: 0.68-0.96, *p* trend <0.0001), maintaining statistical significance only for total mortality. The HR for total mortality was lower when 3-4 servings were consumed daily; HR: 0.78; 95% CI: 0. 69-0.88, and did not improve with a higher consumption. When we analyzed the intake of these foods separately, fruit consumption had the greatest association with lower CVD, total mortality and non-CV mortality risk, while the consumption of legumes was inversely associated with non-CV death and total mortality. The consumption of raw vegetables was strongly associated with lower risk of total mortality, while the consumption of cooked vegetables showed only a small benefit in total mortality. In conclusion, a high consumption of fruits, vegetables and legumes are associated with lower risk of total mortality and non-CV mortality. the benefits are maximum with 3-4 daily servings, equivalent to 375-500 g/day.

## Impact of consumption and substitutions of macronutrients on lipid profile and blood pressure

In the third article ^22^, we reported the results of the analysis of the influence of food consumption on intermediate markers of cardiovascular risk such as lipid profile and blood pressure. The association between macronutrients and dietary cholesterol with risk markers for CVD was evaluated using multilevel modelling. The effect of isocaloric replacement of saturated fat with other fats and with carbohydrates was determined globally and by levels of intake, using nutrient density models. In the simulation model, we assumed that the effects of saturated fat on CV events were related only to an individual risk marker, then we compared these simulated risk markers with the associations observed directly between saturated fat and CV events. In the analysis, we found that the total intake of fat, as well as the individual intake of each type of fat was associated with a higher concentration of total cholesterol and LDL-cholesterol, but also with higher levels of HDL-cholesterol and apolipoprotein A1 (ApoA1) and lower levels of Triglycerides, Total Cholesterol / HDL-cholesterol ratio, Triglycerides / HDL cholesterol ratio and the apolipoprotein B (ApoB) / ApoA1 ratio (all with a *p* trend <0.0001). The highest intake of carbohydrates was associated with the lowest levels of total cholesterol, LDL-cholesterol and ApoB, but also with lower levels of HDL-cholesterol and ApoA1, and higher levels of triglycerides, total cholesterol / HDL-cholesterol ratio, Triglycerides / HDL-cholesterol ratio, and the ApoB / ApoA1 ratio (all p trends <0.0001). In the INTERHEART ^26^ and INTERSTROKE ^27^ studies, the ApoB / ApoA1 ratio was the lipid marker that was most strongly associated with AMI and stroke risk.

Higher intakes of total fat, saturated fat and carbohydrates were associated with higher blood pressure levels, while higher protein intake was associated with lower blood pressure levels. This is consistent with the data reported in the INTERMAP study ^28^, a cross-sectional epidemiological study conducted in the United States, Japan, the United Kingdom and China, in which an inverse relationship was observed between the blood pressure values and the consumption of vegetable proteins, total and insoluble fiber, polyunsaturated fatty acids, total n-3 fatty acids and linoleic acid, glutamic acid, phosphorus, calcium, magnesium, and non-heme iron; and there was a direct relationship between blood pressure and the consumption of sugars and sugary drinks, cholesterol, glycine, alanine and oleic acid from animal sources.

The replacement of saturated fat with carbohydrates was associated with the largest adverse effects on the lipid profile, while the replacement of saturated fat with unsaturated fat improves some risk markers (LDL-cholesterol, blood pressure), but negatively affects others such as HDL- cholesterol and triglycerides. The association between saturated fat and fewer CV events was related to the simulated association mediated by the effects on the ApoB / ApoA1 ratio, but not with other lipid markers, including LDL-cholesterol.

Taken together, our data contradict current recommendations to reduce saturated fat intake and replace it with carbohydrates, an action that has an adverse effect on the lipid profile; Substituting saturated fat with unsaturated fat may improve some risk markers, but may adversely affect others. The simulations carried out suggest that the ApoB / ApoA1 ratio is among all the lipid markers, the best marker of the effect of saturated fat on the risk of CVD, a result that is of great importance for public health, because it means that focus the evaluation of CVD risk of a single lipid marker, such as LDL-cholesterol, does not allow the evaluation of the net clinical effects of nutrient intake on cardiovascular risk.

In conclusion, our results show that to evaluate the CVD risk it is important to consider in addition to LDL-cholesterol, the other fractions of cholesterol, especially when determining the net effect of the substitution of one macronutrient for another. Our findings also show that the moderate intake of saturated fat is more favourable than the excessive intake of carbohydrates. Demonstrating that the consumption of approximately 10% of the total daily energy consumed as saturated fat is more favourable for cardiovascular health than an excessive consumption of carbohydrates (more than 65% of the total energy consumed daily) is in agreement with reports of studies conducted in high-income countries in which it was shown that replacing saturated fat with carbohydrates does not produce any beneficial effect in terms of reducing the risk of CVD ^9^.

As we consider the results of the PURE study as a whole, we can conclude that focusing attention on a single risk marker for lipids, LDL-cholesterol case, is not correct and that preventive nutritional actions should have as a key priority the reduction of carbohydrate intake, especially of processed and ultra-processed carbohydrates, as a higher priority than that of total fat and saturated fat. The clinical implications of these results in clinical practice, as well as public health policies, are important and will lead to fundamental changes in dietary guidelines throughout the world, given the international nature of the PURE study.

## Consumption of Andean legumes and risk of cardio-metabolic diseases

A higher consumption of fruits, vegetables and legumes is inversely associated with CVD, AMI, CV mortality, non-CV mortality and total mortality. Although the PURE study, due to its international nature, is applicable to all countries, it does not discriminate the effect of the consumption of specific foods such as the Andean native legumes on LCAs and their potential beneficial effects on the health of the population that inhabits this region of the world.

The legume plants considered as typical Andean include varieties of grains that are harvested dry such as peas, lentils, beans, beans, chochos / tarwi ^29^. This group of grains does not include peanuts or soybeans because of their high oil content. These legumes are rich in proteins, folate, iron, potassium, zinc and fibber, nutrients whose consumption are inversely related to blood pressure levels ^28^ and which differentiate them from other foods such as cereals ^29^ ^,^ ^30^. Due to the important nutritional characteristics of legumes, they are recommended as an important component of the diet by the food guides of most countries that have guidelines ^31^ promoted around the world by the United Nations, an entity that declared 2016 as the year of legumes. There is no consensus on the recommended amount of legume consumption for a balanced and healthy diet, which may have limited its promotion and consumption at a population level. Recent international studies on the consumption of legumes indicate that consuming 100 g of cooked legumes, two or three times a week, help to ameliorate deficiencies of nutrient contained in these foods ^32^. The consumption of these foods is associated with a reduction in the risk of developing chronic noncommunicable diseases ^33^ ^,^ ^34^. These data indicate that legumes are highly nutritious, and are also reasonably priced foods consumed throughout the world which have positive effects on health.

Next, we review the existing evidence on legume consumption and its association with total, non-cardiovascular and cardiovascular mortality. The results of the PURE study ^21^ show that the high consumption of legumes was inversely associated with cardiovascular mortality, non-cardiovascular mortality and total mortality in the minimally adjusted models, and with non-cardiovascular mortality and total mortality in the fully adjusted models. A meta-analysis of six prospective studies involving 218,997 people showed that a high consumption of legumes was associated with a significant decrease in all-cause mortality, but did not find a significant reduction in cardiovascular mortality ^31^. In another similar analysis, legume consumption of approximately 150 g/day was associated with a significant 16% reduction in all-cause mortality ^32^. A systematic review and meta-analysis study involving 367,000 individuals with 18,475 cases of cardiovascular events (7,451 acute myocardial infarction and 6,336 cases of cerebral infarction) demonstrated that high legume consumption was associated with a significant 10% reduction in cardiovascular mortality and acute myocardial infarction. When the consumption of legumes and cerebral infarction was analyzed separately, no significant relationship was found ^34^, however the evidence shows that the consumption of legumes is beneficial to reduce all-cause mortality ^35^. We believe that currently, studies are still necessary that consider the type, quantity, optimal intake and frequency of consumption of legumes and their relationship with cardio-metabolic diseases. Evidence suggests that the consumption of legumes has a protective effect on cardiovascular mortality ^21^ ^,^ ^34^ ^-^ ^37^. It is necessary to continue documenting the relationship between cardio-metabolic diseases and the consumption of legumes since it is possible that characteristics of each legume could have different effects on metabolic pathology.

The plants of the genus *Lupinus* are legumes rich in proteins, fats, complex carbohydrates, and micronutrients that have for centuries been consumed in the Middle East, the Mediterranean Region and the American Andes, a region in which the lupine variety that predominates is *L Mutabilis* ^39^. Studies with animal models of obesity and diabetes, as well as studies in individuals with risk factors for CVD (hypertension, dyslipidemia, dysglicemia, metabolic syndrome) have shown that the administration of lupinus has positive effects on blood pressure ^37^, the lipid profile ^40^ ^,^ ^41^ and blood glucose levels ^42^ ^,^ ^43^. The mechanisms responsible for the beneficial effects on blood pressure could be due to lupinus peptides generated by their digestion with pepsin, capable of inhibiting the angiotensin converting enzyme ^44^. The beneficial effect of lupinus consumption observed in patients with dyslipidemias could be related to the fiber content of this legume and its metabolites, promoting the production of short-chain fatty acids by the gut microbiota ^45^.

We have shown that consuming *L. mutabilis* improves the sensitivity of the insulin receptor and consequently decreases blood glucose levels in patients with DM2 ^42^ ^,^ ^43^. These studies also showed that the consumption of crude *L. mutabilis* (3.1 mg/kg) by healthy young people did not modify glucose or insulin concentrations, but it did so in individuals with dysglycemia ^42^, while the consumption of cooked *L. mutabilis* (2.5 mg/kg) in patients recently diagnosed with DM2 significantly decreased glucose and insulin concentrations ^43^, demonstrating that the hypoglycemic effect of cooked *L. mutabilis* is greater than the consumption of raw lupinus (14.3.25% vs 5.9%) ^43^. the consumption of cooked *L. mutabilis* did not cause adverse effects, and is important component of the diet in the Andean region, mainly in Bolivia, Ecuador, and Peru, where the consumption of *L. mutabilis* de-bittered by cooking is common and well accepted.

In recent years, the components of the Lupinus grains responsible for the improvement of glucose metabolism have been identified. Bertoglio *et al*. ^46^, demonstrated a hypoglycemic effect with increasing doses of preparations enriched with the γ-conglutin protein derived from *L. albus* grains in rats and in humans. In this study, an immediate progressive hypoglycemic effect was observed in 15 volunteers who were given 75 g of carbohydrates and treated with increasing doses of γ-conglutin (from 750 to 3,000 mg) ^46^. In an experimental study, chronic treatment of γ-conglutin in rats with hyperglycemia reduced glucose and insulin levels. Thus, the simultaneous administration of glucose (2-3 g / d) and γ-conglutin (28 mg/kg body weight) limited the increase in plasma glucose and insulin concentrations ^47^. In addition, chronic treatment with γ-conglutin in rats with diabetes decreased glucose concentrations and the expression of the enzyme gene glucose-6-phosphatase (G6pc) at the liver level, an enzyme that is responsible for the control of gluconeogenesis ^48^. It has also been shown that treatment with γ-conglutin in rats with diabetes increases the expression of insulin at the protein and mRNA level ^48^ ^,^ ^49^. Taken together these data indicate that derivatives of *L. albus* have favorable effects on glucose and insulin metabolism, however the bioactive components of the Andean *L. mutabilis* responsible for the decrease in blood glucose in people with diabetes are not known. Recent studies in our group indicate that peptides derived from the de-fatted *L. mutabilis* grain will lead to inhibition of the enzyme DPP-IV, an increase in the sensitivity of the insulin receptor, an increase in the number of glucose transporters in the membrane adipocyte cell and an inhibition of hepatic gluconeogenesis ^50^.

## Conclusions

The scientific evidence reviewed shows that a healthy diet must be balanced and varied, be composed of an adequate proportion of complex carbohydrates rich in fibre between 50-55% of the daily energy consumed, of saturated and unsaturated fat (25-30%), animal and vegetable protein (including legumes) between 15-25%, vitamins, minerals and water. These nutrients are abundantly present in fruits, vegetables, cereals, legumes, milk and its derivatives, eggs and meats. Public policies must consider this new scientific evidence to promote primary prevention programs that contribute to reducing the growing prevalence of cardio-metabolic diseases, as well as helping in the treatment of these diseases once established. It is also necessary to spread the benefits of consuming Andean legumes that have been traditional in the peoples that inhabit this region of the world. 
