# Supplementary material for: Reevaluating nutrition as a risk factor for cardio-metabolic diseases
Source: Colomb Med (Cali). 2018 Jun 30;49(2):175–81. doi: 10.25100/cm.v49i2.3840 (PMC6084918; doi:10.25100/cm.v49i2.3840)
Supplement: Related texts

# Other: Reevaluando la nutrición como factor de riesgo para las enfermedades cardio-metabólicas

## Introducción

El concepto oficial de que las grasas saturadas causan enfermedad coronaria viene desde 1961 cuando la Asociación Americana del Corazón (AHA) publicó la primera recomendación de evitar el consumo de grasas saturadas, así como del colesterol con el objetivo de prevenir las Enfermedades Cardiovasculares (ECV) ^1^. Esta propuesta es conocida como la “hipótesis de la relación dieta-corazón” y apareció como una explicación necesaria para entender el riesgo de padecer estas enfermedades que son de gran interés tanto en salud pública como en la práctica clínica dado el incremento de su incidencia en los últimos años, Sin embargo, la causalidad de esta hipótesis falta ser comprobada. 

las ECV son la primera causa de mortalidad en el mundo entero, incluso en los países de ingresos medianos y bajos tales como Colombia y Ecuador ^2^. En Colombia, de acuerdo a los datos del *Institute for Health Metrics and Evaluation*, la enfermedad isquémica coronaria se mantiene como la primera causa de mortalidad, con un incremento del 21.26% desde el 2005 al 2016, mientras que la mortalidad por accidente cerebro-vascular (ACV) se ha mantenido estable con una tendencia mínima a disminuir (-1.3%) ^3^. La importancia que este tema tiene en las políticas de salud pública y en la práctica clínica, ha dado lugar a un debate con argumentos controversiales, en la literatura de difusión científica y en artículos de divulgación científica como la publicación de Teicholz y Thorn ^4^, artículo del cual utilizamos a continuación algunas de las argumentaciones expuestas por estos autores. 

El objetivo de la presente revisión, particularmente de los datos del estudio “Prospective Urban and Rural Epidemiology” (PURE), consiste en demostrar que una dieta saludable debe ser equilibrada y variada, estar compuesta con una adecuada proporción de carbohidratos complejos ricos en fibra entre 50-55% de la energía diaria consumida, de grasa saturada e insaturada (25-30%), de proteína animal y vegetal (incluidas las leguminosas) entre 15- 25%, de vitaminas, minerales y agua, y que por lo tanto las recomendaciones de dietas muy restrictivas en alimentos ricos en colesterol y grasas saturadas, de frente al excesivo consumo de carbohidratos especialmente procesados, deben ser reevaluadas, ya que el consumo de alimento ricos en colesterol tienen un impacto en los niveles plasmáticos de LDL-colesterol, sino también sobre las concentraciones HDL-colesterol, triglicéridos y apolipoproteínas, y que estudios en los que se ha sustituido el consumo de ácidos grasos saturados por ácidos grasos monoinsaturados o poliinsaturados procedentes de aceites vegetales no han demostrado consistentemente un efecto beneficioso sobre la incidencia de eventos cardiovasculares y sobre el desarrollo de diabetes mellitus tipo 2 (DM2), que ha efectos de esta revisión la denominamos como enfermedades cardio-metabólicas. 

## Estudios de sustitución de grasas saturadas por aceites vegetales 

Los autores anteriormente citados ^4^ afirman que durante las décadas de los 60-80 se invirtieron billones de dólares en grandes estudios clínicos que incluyeron entre 10,000 y 53,000 individuos, en los que se probaron diferentes dietas en las que se sustituyeron las grasas saturadas con aceites vegetales ricos en grasas insaturadas; sin embargo, los resultados no fueron los esperados para respaldar el propuesto papel causal de las grasas saturadas en la presentación de ECV. Argumentan los autores citados que muchos de estos resultados fueron poco difundidos, y que inclusive uno de los más grandes ensayos clínicos, financiado por los Institutos Nacionales de Salud de los Estados Unidos (INH), obtuvo resultados contrarios a los esperados efectos beneficiosos de la sustitución de las grasas saturadas por los aceites ricos en grasa insaturada, motivo por el cual no fueron publicados durante más de 16 años ^5^. 

Recientemente, varias publicaciones realizadas en los Estados Unidos de América revisaron los resultados de algunos ensayos clínicos ^6^^,^^7^ que fueron condenados al olvido o fueron reanalizados ^7^. En ninguna de las revisiones realizadas y en los meta-análisis publicados sobre este tema, se ha podido documentar evidencia de que las grasas saturadas tuvieran un efecto negativo en la mortalidad total o en la mortalidad cardiovascular ^8^^,^^16^. A pesar de estas evidencias, las guías dietéticas de diferentes países, que basan sus recomendaciones en las guías de USA, indican que el consumo de grasas saturadas debe ser menor al 10% de las calorías diarias consumidas. Las recomendaciones de la AHA son aún más restrictivas, limitando el consumo de grasas saturadas a solo 5-6% de las calorías diarias que deben ser ingeridas por individuos con niveles altos de colesterol en sangre ^17^^,^^18^.

El Consejo Presidencial de la AHA en su más reciente publicación ^19^ concluyó que sustituir las grasas saturadas por ciertos aceites vegetales (soya, girasol) reduce el riesgo de ECV en aproximadamente 30%, reducción que solo es similar a la reportada en ensayos clínicos que utilizaron estatinas. Sin embargo, Teicholz y Thorn ^4^ destacan que estos resultados son contrarios a los de cuatro revisiones realizadas por diferentes autores ^9^^-^^13^, en las cuales la reducción estimada del riesgo de ECV no alcanzó el 19 %, además, que en dos de estas revisiones los resultados perdieron significancia estadística cuando los autores utilizaron criterios más rígidos y no incluyeron en el análisis los ensayos clínicos pobremente controlados. Según los autores citados ^4^ si se examinan solo los resultados con significación estadística de estudios clínicos bien controlados, solo dos revisiones concuerdan con los resultados obtenidos por la AHA, mientras todos los otros análisis arrojan resultados contrarios. La explicación a estas contradicciones en publicaciones que han realizado los análisis de los mismos trabajos en forma independiente, según Teicholz y Thorn ^4^ pueden explicarse por los criterios de los desenlaces utilizados, así, en siete revisiones que utilizaron desenlaces duros como son infarto agudo de miocardio (IAM), accidente cerebrovascular (ACV), muerte cardiovascular o mortalidad total, se encontró que al sustituir las grasas saturadas por aceites vegetales ricos en ácidos grasos polinsaturados no hubo ningún efecto beneficioso. Cuando se utilizó, como lo hace la AHA, un desenlace más blando, como es el compuesto de varios eventos CV, entre los que se incluyen eventos más subjetivos como angina, se encuentra un pequeño efecto beneficioso de la sustitución, así como también si en el análisis se seleccionaron solo algunos estudios. En verdad, según Teicholz y Thorn ^4^ existen argumentos y evidencias que ponen en entredicho la hipótesis de la relación dieta-corazón.

## Contribuciones del estudio PURE para clarificar la participación del consumo de macronutrientes en el riesgo cardiovascular

De frente a la serie de contradicciones en las publicaciones mencionadas, en el estudio PURE evaluamos el impacto de la composición de la dieta en ciertos factores de riesgo como perfil lipídico, presión arterial y en el riesgo de ECV ^20^^-^^22^. Más de 135,000 individuos de 18 países de ingresos económicos altos (PIA), medianos (PIM) y bajos (PIB) de cinco continentes, fueron seguidos durante 7.4 años en promedio, en los cuales estudiamos la asociación del consumo de macro-nutrientes con la morbilidad y mortalidad cardiovascular. En el primer reporte ^20^, demostramos que un mayor consumo de grasas (saturadas, monoinsaturadas y poliinsaturadas) y de proteínas de origen animal, se asoció con una menor tasa de mortalidad, en tanto que el mayor consumo de carbohidratos se asoció con una aumentada tasa de mortalidad. Los desenlaces primarios estudiados fueron mortalidad de cualquier causa y eventos cardiovasculares mayores: muerte cardiovascular, IAM no fatal, ACV no fatal y falla cardíaca. Los desenlaces secundarios fueron todos los infartos cardiacos, ACV, mortalidad cardiovascular y mortalidad no cardiovascular. Los participantes fueron categorizados por quintiles de ingesta de nutrientes: carbohidratos, grasas y proteínas, basados en el porcentaje de energía proporcionado por cada macronutriente. Evaluamos la asociación entre consumo de carbohidratos, grasa total y cada tipo de grasa con ECV y mortalidad total. Calculamos los cocientes de riesgo (HR) usando un modelo de riesgos proporcionales de Cox. Durante el seguimiento, se presentaron 5,796 muertes y 4,784 eventos CV mayores. El mayor consumo de carbohidratos se asoció con un aumento en el riesgo de mortalidad total (mayor quintil 5 vs menor quintil 1) HR de 1.28; IC 95%: 1.12-1.46, *p* trend= 0.0001), pero no con el riesgo de ECV o mortalidad cardiovascular. La ingesta de grasa total y de cada uno de sus tipos se asoció con un menor riesgo de mortalidad por cualquier causa (quintil 5 vs quintil 1 de grasa total: HR 0.77; IC 95%: 0.67-0.87, *p* trend <0.0001; de grasa saturada: HR 0.86; IC 95%: 0.76-0.99, *p* trend= 0.0088; de grasa monoinsaturada: HR 0.81; IC 95%: 0.71-0.92, *p* trend <0.0001; y de grasa poliinsaturada: HR: 0.80; IC 95%: 0.71-0.89, *p* trend <0.0001). Una mayor ingesta de grasa saturada se asoció con un menor riesgo de ACV (quintil 5 vs quintil 1, HR 0.79; IC 95 %: 0.64-0.98, *p* trend= 0.0498). El consumo de grasa total, saturada e insaturada no se asoció con riesgo de IAM o mortalidad por ECV. En conclusión, nuestros hallazgos demuestran que la alta ingesta de carbohidratos es la que se asocia con mayor riesgo de mortalidad total, mientras que el consumo de grasa total o de sus diferentes tipos se asocia con menor mortalidad por cualquier causa y no se asocia con ECV, IAM o mortalidad CV. Además, el consumo de grasa saturada tiene una asociación inversa con ACV.

Estos resultados demuestran que en la población estudiada, la mayoría provenientes de PIM y PIB, existe una relación inversa entre consumo de carnes y leche (que fueron la mayor fuente de proteínas y grasas saturadas y monoinsaturadas) con la mortalidad por cualquier causa. , este resultado puede estar relacionado con el hecho de que los productos animales son ricos en zinc, hierro, vitamina K2 y vitamina B12, micronutrientes que son deficientes en las poblaciones de PIM y PIB. ^23^, En estos países, existe un bajo consumo de productos animales por ser caros e inaccesibles ^24^, lo que determina que exista un alto consumo de carbohidratos en forma compensatoria, que como se ha indicado, se asocian a una mayor mortalidad. También, podría deberse a que una alimentación con un adecuado aporte de proteínas y grasas, prevendría la sarcopenia y la pérdida de fuerza muscular asociadas al envejecimiento, que son factores de riesgo para ECV como se demostró por el mismo estudio PURE ^25^.

## Consumo de frutas, verduras y leguminosas y riesgo cardiovascular

En el segundo artículo ^21^, reportamos que la ingesta adecuada de frutas, leguminosas y verduras crudas, alimentos que son ricos en carbohidratos complejos, se asocia con una menor mortalidad global. Esta aparente discrepancia sugiere que es el consumo alto de carbohidratos procesados (azúcar y harinas refinadas) el que se asocia con la mayor mortalidad observada en los individuos estudiados. Para evaluar el consumo utilizamos un cuestionario de frecuencia alimentaria específico para cada país y cuestionarios estandarizados para colectar información sobre variables demográficas (edad, sexo), socio-económicas (educación, ingreso, empleo), estilos de vida (tabaquismo, actividad física, ingesta de alcohol), antecedentes clínicos, uso de medicamentos e historia familiar de ECV. El desenlace principal fue presencia de ECV y al igual que en la publicación anterior, empleamos los mismos análisis estadísticos para evaluar la asociación entre consumo de frutas, vegetales y legumbres con el riesgo de eventos CV y mortalidad total. El consumo promedio de frutas, vegetales y legumbres fue de 3.91 (DE 2.77) porciones por día. Un mayor consumo total de frutas, verduras y legumbres se asoció inversamente con ECV, IAM, mortalidad CV, mortalidad no CV y mortalidad total, en el modelo ajustado por edad, sexo y centro médico. Los estimados fueron atenuados en el modelo multivariado ECV (HR: 0.90; IC 95%: 0.74-1.10, *p* trend= 0.1301), IAM (HR: 0.99; IC 95%: 0.74-1.31; *p* trend=0.2033), ACV (HR: 0.92, IC 95%: 0.67-1.25; *p* trend= 0.7092), mortalidad CV (HR: 0.73, IC 95%: 0.53-1.02; *p* trend= 0.0568), mortalidad no CV (HR: 0.84; IC 95%: 0.68-1.04; *p* trend= 0.0038), y mortalidad total (HR: 0.81, IC 95%: 0.68-0.96; *p* trend <0.0001), manteniéndose la significación estadística solamente para la mortalidad total. El HR para mortalidad total fue más bajo cuando se consumen 3-4 porciones diarias (HR: 0.78; IC 95%: 0.69-0.88) y no mejoró con un consumo mayor. Cuando analizamos separadamente la ingesta de estos alimentos, el consumo de frutas fue el que más se asoció con menor riesgo de ECV, mortalidad total y mortalidad no CV, mientras que el consumo de legumbres está inversamente asociado con muerte no CV y mortalidad total. El consumo de vegetales crudos estuvo fuertemente asociado con menor riesgo de mortalidad total, mientras que el consumo de vegetales cocidos solo mostró un pequeño beneficio en la mortalidad total. En conclusión, un alto consumo de frutas, vegetales y legumbres se asocian con menor riesgo de mortalidad total y mortalidad no CV. los beneficios son máximos con 3-4 porciones diarias, equivalentes a 375-500 g/día.

## Impacto del consumo y sustituciones de macronutrientes en perfil lipídico y presión arterial

En el tercer artículo ^22^, reportamos los resultados del análisis de la influencia del consumo de alimentos en marcadores intermedios de riesgo cardiovascular como son el perfil lipídico y la presión sanguínea. La asociación entre los macronutrientes y el colesterol dietético con los marcadores de riesgo para ECV fueron evaluados utilizando un modelamiento multinivel. El efecto del reemplazo isocalórico de grasa saturada con otras grasas y carbohidratos fue determinado globalmente y por niveles de ingesta, usando modelos de densidad de nutrientes. En el modelo de simulación asumimos que los efectos de la grasa saturada sobre los eventos CV estuvieron relacionados únicamente con un marcador de riesgo individual, luego comparamos estos marcadores de riesgo simulados con las asociaciones observadas directamente entre grasa saturada y eventos CV. En el análisis, encontramos que la ingesta total de grasa, así como la ingesta individual de cada tipo de grasa se asocia con una mayor concentración de colesterol total y LDL-colesterol, pero también con mayores niveles de HDL-colesterol y apolipoproteina A1 (ApoA1) y menores niveles de Triglicéridos, de la proporción Colesterol total/HDL-colesterol, de la proporción Triglicéridos/HDL colesterol y de la proporción de apolipoproteína B (ApoB)/ApoA1 (todos con un *p* trend <0.0001). La mayor ingesta de carbohidratos se asoció con menores niveles de colesterol total, LDL-colesterol y ApoB, pero también con menores niveles de HDL-colesterol y ApoA1, y mayores niveles de triglicéridos, de la proporción Colesterol total/HDL-colesterol, de la proporción Triglicéridos/HDL-colesterol, y de la proporción ApoB/ApoA1 (todos *p* trend <0.0001). en los estudios INTERHEART ^26^ e INTERSTROKE ^27^ la relación ApoB/ApoA1 fue el marcador lipídico de riesgo que mejor se asoció con IAM y ACV. 

La mayor ingesta de grasa total, grasa saturada y carbohidratos se asociaron con niveles más altos de presión sanguínea, mientras que un mayor consumo de proteínas se asoció con menores cifras de presión sanguínea. Esto concuerdan con los datos reportados en el estudio INTERMAP ^28^, estudio epidemiológico transversal realizado en Estado Unidos, Japón, Reino Unido y China, en el que se observó una relación inversa entre las cifras de presión arterial con el consumo de proteínas vegetales, fibra total e insoluble, ácidos grasos polinsaturados, ácidos grasos n-3 totales y ácido linoleico, ácido glutámico, fosforo, calcio, magnesio, y hierro no hemínico con; así como una relación directa de la presión sanguínea con el consumo de azucares y de bebidas azucaradas, colesterol, glicina, alanina y ácido oleico de fuentes animales. 

El reemplazo de grasa saturada con carbohidratos se asoció con los mayores efectos adversos en el perfil lipídico, mientras que el reemplazo de la grasa saturada por grasa insaturada mejora algunos marcadores de riesgo (LDL-colesterol, presión sanguínea), pero afecta otros como HDL-colesterol y triglicéridos. La asociación entre grasa saturada y menos eventos CV estuvo relaciona con la asociación simulada mediada por los efectos en la proporción de ApoB/ApoA1, pero no con otros marcadores lipídicos, inclusive LDL-colesterol. 

En su conjunto, nuestros datos contradicen las recomendaciones actuales de reducir la ingesta de grasa saturada y reemplazarla con carbohidratos, acción que tiene un efecto adverso en el perfil lipídico; sustituir la grasa saturada con grasa insaturada puede mejorar algunos marcadores de riesgo, pero puede afectar otros. Las simulaciones realizadas sugieren que la proporción ApoB/ApoA1 es de entre todos los marcadores lipídicos, el mejor marcador del efecto de la grasa saturada en el riesgo de ECV, resultado que tiene una gran importancia para la salud pública, porque significa que centrar la evaluación del riesgo de ECV solo en un marcador lipídico, como el LDL-colesterol, no permite evaluar los efectos clínicos netos de la ingesta de nutrientes en el riego cardiovascular.

Estos resultados demuestran que para evaluar el riesgo de ECV es importante considerar las otras fracciones del colesterol, además del LDL-colesterol, especialmente cuando se considera el efecto neto de la sustitución de un macronutriente por otro. También, demuestran que la ingesta de grasa saturada es más favorable que la excesiva ingesta de carbohidratos. La demostración que el consumo de la grasa saturada en porcentajes de alrededor del 10 % de la energía diaria total consumida es más favorable para la salud cardiovascular que el consumo excesivo de carbohidratos (más del 65% de la energía total consumida diariamente) está en concordancia con reportes de estudios realizados en países de altos ingresos en los que se demostró que reemplazar la grasa saturada con carbohidratos no produce ningún efecto beneficioso en términos de reducir el riesgo de ECV ^9^. Desafortunadamente, la calidad de los carbohidratos no fue evaluada en el estudio PURE; sin embargo, el hecho de que el consumo de carbohidratos provenientes de frutas, vegetales y legumbres tengan un efecto beneficiosos sobre el riesgo de ECV, sugieren que el riesgo es dado por el alto consumo de carbohidratos refinados. 

Los resultados del estudio PURE demuestran que el centrar la atención en un solo marcador de riesgo para los lípidos, caso LDL-colesterol, no es correcto y que las acciones nutricionales preventivas deben tener como prioridad clave la reducción de la ingesta de carbohidratos, especialmente de los carbohidratos procesado y ultraprocesados, antes que de la de la grasa total y de la grasa saturada. Las implicaciones clínicas de estos resultados en la práctica clínica, así como para las políticas de salud pública, son importantes y darán lugar a cambios fundamentales en las guías dietéticas en todo el mundo, dado el carácter internacional del estudio PURE.

## Consumo de leguminosas andinas y riesgo de enfermedades cardio-metabólicas

El mayor consumo de frutas, verduras y legumbres está asociado inversamente con ECV, IAM, mortalidad CV, mortalidad no CV y mortalidad total. Si bien el estudio PURE por su carácter internacional es aplicable a todos los países, no permite discriminar el efecto que sobre las ECV tiene el consumo de leguminosas típicamente andinas y de su efecto benéfico en la salud de la población que habita esta región del mundo.

Las plantas leguminosas consideradas como típicamente andinas incluyen variedades de granos que son cosechados secos como las arvejas, lentejas, frijoles, habas, chochos/tarwi ^29^. En este grupo de granos no se incluye al maní o a la soya por sus contenidos altos de aceite. Estas leguminosas son alimentos ricos en proteínas, folato, hierro, potasio, zinc y fibra, nutrientes cuyo consumo se relacionan de forma inversa con los niveles de presión arterial ^28^ y que las diferencian de otros alimentos como los cereales ^29^^,^^30^, por lo que son recomendadas por las guías alimentarias de la mayoría de países que poseen estas guías. ^31^. Por las importantes características nutricionales de las leguminosas, su consumo es promocionado alrededor del mundo por las Naciones Unidas, entidad que declaró al 2016 como el año de las leguminosas. No existe consenso sobre la cantidad recomendada del consumo de leguminosas para una dieta balanceada y saludable, lo que ha limitado su promoción y consumo a nivel general. Estudios recientes sobre el consumo de leguminosas a nivel mundial indican que consumir 100 g de leguminosas cocidas, dos o tres veces por semana contribuyen a mejorar las deficiencias de nutrientes contenidos en estos alimentos ^32^. Existe evidencia de que el consumo de estos alimentos está asociado con una disminución del riesgo de desarrollar enfermedades crónicas no transmisibles ^33^^,^^34^. Estos datos indican que las leguminosas son alimentos altamente nutritivos, de costo razonable, que se consumen en todo el mundo y que tienen efectos positivos para la salud. 

A continuación revisamos la evidencia existente sobre consumo de leguminosas y su asociación con mortalidad total, no cardiovascular y cardiovascular. Los resultados del estudio PURE ^21^ demuestran que el consumo alto de leguminosas se asoció inversamente con mortalidad cardiovascular, mortalidad no cardiovascular y mortalidad total en los modelos mínimamente ajustados, y con mortalidad no cardiovascular y mortalidad total en los modelos completamente ajustados. Un metaanálisis de seis estudios prospectivos que incluyeron 218,997 personas demostró que un alto consumo de leguminosas se asoció con una disminución significativa de mortalidad por todas las causas, pero no encontró una reducción significativa de la mortalidad cardiovascular ^31^. En otro análisis similar, el consumo de leguminosas de aproximadamente 150 g/día estuvo asociado con una reducción significativa del 16 % en la mortalidad por todas las causas ^32^. Un estudio de revisión sistemática y metaanálisis que incluyó 367,000 individuos con 18,475 casos de eventos cardiovasculares (7,451 infarto agudo de miocardio y 6,336 de infarto cerebral) demostró que el consumo alto de leguminosas se asoció con una reducción significativa del 10% en mortalidad cardiovascular y de infarto agudo de miocardio. Cuando se analizó separadamente el consumo de leguminosas y el infarto cerebral no se encontró una relación significativa ^34^, sin embargo las evidencias demuestran que el consumo de leguminosas es beneficioso para reducir la mortalidad por todas las causas ^35^. Creemos que al momento todavía son necesarios estudios que consideren el tipo, la cantidad, la ingesta óptima y la frecuencia del consumo de las leguminosas y su relación con las enfermedades cardio-metabólicas. La evidencia sugiere que el consumo de leguminosas tiene un efecto protector sobre la mortalidad cardiovascular ^21^^,^^34^^-^^37^. Se requiere seguir documentando la relación entre enfermedades cardio-metabólicas y el consumo de leguminosas ya que es posible que características propias de cada leguminosa pudieran tener efectos distintos en la patología metabólica.

Se ha propuesto que el consumo de alimentos ricos en proteínas animales, como la leche y sus derivados; y vegetales, como las leguminosas, podría mejorar la homeostasis de la glucosa y la sensibilidad insulina. Estudios clínicos realizados en pacientes con DM2 en quienes se suplementó con proteínas usuales de la dieta, muestran que péptidos bioactivos producto de su digestión, mejoran el metabolismo de la glucosa y de la insulina mediante la inhibición de enzimas como la alfa-glucosidasa y la dipeptidil-dipeptidasa IV (DPP-IV) ^38^. 

Las plantas del género Lupinus son leguminosas ricas en proteínas, grasas, carbohidratos complejos, y micronutrientes que tradicionalmente se han consumido por siglos en Medio Oriente, la Región Mediterránea y en los Andes Americanos, región en la cual la variedad de lupinus que predomina es *L. mutabilis* ^39^. Estudios con modelos animales de obesidad y diabetes, así como también estudios en individuos con factores de riesgo para ECV (hipertensión, dislipidemia, disglicemia, síndrome metabólico) han demostrado que la administración de lupinus tiene efectos positivos en la presión sanguínea ^37^, el perfil lipídico ^40^^,^^41^ y los niveles de glucosa en sangre ^42^^,^^43^. Los mecanismos responsables de los efectos benéficos sobre la presión arterial podrían deberse a péptidos de lupinus generados por su digestión con pepsina capaces de inhibir a la enzima conversora de angiotensina ^44^. El efecto benéfico del consumo de lupinus observado en pacientes con dislipidemias podría estar relacionado con el contenido de fibra de esta leguminosa y de su metabolismo para la producción de ácidos grasos de cadena corta por la microbiota intestinal ^45^.

Nosotros hemos demostrado que consumir el *L. mutabilis* mejora la sensibilidad del receptor de la insulina y consecuentemente disminuye los niveles de glucosa en sangre en pacientes con DM2 ^42^^,^^43^. Estos estudios también demostraron que el consumo de *L. mutabilis* crudo (3.1 mg/kg) por jóvenes saludables no modificó las concentraciones ni de glucosa ni de insulina, en tanto que en personas con disglicemia disminuyó las concentraciones séricas de glucosa e insulina ^42^, mientras que el consumo de *L. mutabilis* cocido (2.5 mg/kg) a pacientes recientemente diagnosticadas de DM2 disminuyó significativamente las concentraciones de glucosa e insulina ^43^, lo que demuestra que el efecto hipoglucemiente de *L. mutabilis* cocido es mayor que el consumo de lupinus crudo (14.3% vs 5.9%) ^43^. el consumo de *L. mutabilis* cocido no causó efectos adversos, y es importante para la población de la región Andina, principalmente en Bolivia, Ecuador, y Perú, donde el consumo de *L. mutabilis* desamargado por cocción es común y bien aceptado.

En los últimos años se han identificado los componentes de los granos de Lupinus responsables del mejoramiento del metabolismo de la glucosa, así, Bertoglio *et al*. ^46^, demostraron un efecto hipoglicemiante con dosis crecientes de preparados enriquecidos con la proteína γ-conglutina derivada de granos de *L. albus* en ratas y en humanos. En ese estudio se observó un efecto hipoglicemiante progresivo inmediato en 15 voluntarios a los que se les administró 75 g de carbohidratos y se los trató con dosis crecientes de γ-conglutina (desde 750 a 3,000 mg) ^46^. En un estudio experimental el tratamiento crónico de γ-conglutina en ratas con hiperglicemia redujo los niveles de glucosa e insulina. Así, la administración simultánea de glucosa (2-3 g/d) y γ-conglutina (28 mg/kg de peso corporal) limitó el aumento de las concentraciones plasmáticas de glucosa e insulina ^47^. Además, el tratamiento crónico con γ-conglutina a ratas con diabetes disminuyó las concentraciones de glucosa y la expresión del gen de la enzima glucosa-6-fosfatasa (*G6pc*) a nivel hepático, enzima que es responsable del control de la gluconeogénesis ^48^. También se ha demostrado que el tratamiento con γ-conglutina a ratas con diabetes aumenta la expresión de la insulina a nivel proteico y de RNAm ^48^^,^^49^. En conjunto estos datos indican que derivados de *L. albus* tienen efectos favorables en el metabolismo de la glucosa y de la insulina, sin embargo no se conocían los componentes bioactivos del *L. mutabilis* andino responsables de la disminución de la glucosa sanguínea en personas con diabetes. Estudios recientes de nuestro grupo indican que péptidos derivados de la harina desengrasada de *L. mutabilis* estarían relacionados con inhibición de la enzima DPP-IV, con aumento de la sensibilidad del receptor de la insulina, con aumento del número de transportadores de glucosa en la membrana celular de adipocitos y con inhibición de la gluconeogénesis hepática ^50^. 

## Conclusiones

La evidencia científica revisada demuestra que una dieta saludable debe ser equilibrada y variada, estar compuesta con una adecuada proporción de carbohidratos complejos ricos en fibra entre 50-55% de la energía diaria consumida, de grasa saturada e insaturada (25-30%), proteína animal y vegetal (incluidas las leguminosas) entre 15-25%, vitaminas, minerales y agua. Estos nutrientes están abundantemente presentes en frutas, vegetales, cereales, leguminosas, leche y sus derivados, huevos y carnes. Las políticas públicas deben considerar esta nueva evidencia científica para promover programas de prevención primaria que contribuyan a disminuir la creciente prevalencia de las enfermedades cardio-metabólicas, así como coadyuvar en el tratamiento de estas enfermedades una vez establecidas. También es necesario la difusión de los beneficios de consumir leguminosas andinas que han sido tradicionales en los pueblos que habitan es esta región del mundo.
